# Effects of Single or Combined Treatments with Radiation and Chemotherapy on Survival and Danger Signals Expression in Glioblastoma Cell Lines

**DOI:** 10.1155/2014/453497

**Published:** 2014-07-01

**Authors:** Francesca Pasi, Alessandro Paolini, Rosanna Nano, Riccardo Di Liberto, Enrica Capelli

**Affiliations:** ^1^Department of Biology and Biotechnology “Lazzaro Spallanzani”, University of Pavia, Via Ferrata 9, 27100 Pavia, Italy; ^2^Department of Medical Physics, IRCCS Policlinico S. Matteo Foundation, Viale Golgi 19, 27100 Pavia, Italy; ^3^Department of Earth and Environmental Sciences, University of Pavia, Via Ferrata 1, 27100 Pavia, Italy

## Abstract

The success of chemo- and radiotherapy in glioblastoma multiforme, the most common and lethal primary brain tumour, could rely on the induction of immunogenic tumour cell death and on the induction of anticancer immune response. In this study we investigated cell survival to single treatments or combination of X-rays and temozolomide in glioblastoma cell lines (T98G and U251MG) and we attempted to identify danger signals (HMGB1 and HSP70) released by dying cells in the microenvironment that could activate antitumour immunity contributing to the therapeutic efficacy of conventional treatments. Our data suggest that HSP70 translocates from cytoplasm to extracellular environment after an increase in radiation dose and HMGB1 translocates from the nucleus to the cytoplasm and subsequently is released into the extracellular space, confirming a role of these proteins as signals released after radiation-induced damage in glioblastoma cells. We also could state that TMZ had limited effectiveness in activating HMGB1 and HSP70 signalling and, instead, an adjuvant effect was observed in some combined treatments, depending on schedule, cell line, and timing. A big challenge in tumour therapy is, therefore, to identify the most beneficial combination and chronology of multiple treatment options to contribute to the improvement of the therapeutic outcome.

## 1. Introduction

Glioblastoma multiforme (GBM, WHO grade IV) is the most common and lethal primary malignant brain tumour that continues to have poor prognosis and a high likelihood of recurrence [1]. The median survival time from the time of diagnosis without any treatment is 3 months and despite the recommended treatment regimen of aggressive surgical resection, radiation, and chemotherapy, it remains approximately 9–11 months [2]. Thus, these tumours continue to present an enormous therapeutic challenge. Since most of the patients develop also a relapse [3], a deeper knowledge is urgently needed in order to find out how the applied therapies can modulate the tumour cells. For this reason, to win the fight against cancer, it is necessary not only to develop strategies to kill all cancer cells efficiently but also to attempt to stimulate an immune response so that the immune system can keep residual tumour cells in check [4]. In particular, combined tumour therapies of radiation and chemotherapy should on the one hand kill the cancer cells and on the other hand induce antigen release and danger signals expression from the tumour. Consequently these signals released in the microenvironment could activate both the innate and adaptive immune system of the host. Hence, the cellular stress induced by treatments dictates the immunological response to dying cells and the activation of immune system might contribute to the therapeutic efficacy of conventional cancer treatments determining synergistic effects of radiation and immunotherapy increases [5].

However, the underlying biological pathways are only partially understood. In particular, the immunogenic potential of different tumour cell types, the number of different danger signals, their molecular identity, their different biological effects, the receptors, and the pathways that sense the release of these signals are still not known. It could be necessary to define their roles in both health and disease and it might be possible to use these molecules, expressed on cell surface or released in the microenvironment, to manipulate immune responses or to inhibit them to treat malignant brain tumour [6].

In the present study, to gain new insights into the mechanisms of radiation and/or chemotherapeutic effect in malignant gliomas we investigated cell survival to single treatment or combination of radiation and temozolomide (TMZ), a chemotherapeutic drug currently used in clinics for the treatments of these tumours. Moreover, to better elucidate target molecules involved in malignant glioma responses, we attempted to identify danger signals like high mobility group box-1 (HMGB1) protein and heat-shock protein 70 (HSP70) in glioblastoma using cell culture system. These proteins are released as danger signals by dying cells and could activate dendritic cells and stimulate antigen processing and presentation to T cells [7]. HSP70 is taken up from dendritic cells for cross-presentation via HSP receptors (e.g., CD91 and CD14) and HMGB1 released by necrotic cells is a potent adjuvant* in vivo* that triggers a protective immune response through activation of TLR4 on DCs [8, 9].

Stimulation by these danger signals, uptake, and presentation of dead tumour cell-derived peptides by mature dendritic cell as well as their consecutive contact with T cells may lead to specific and, most importantly, long-lasting antitumour immunity that might contribute to the therapeutic efficacy of conventional cancer treatments.

For this purpose, glioblastoma cell lines (T98G and U251MG) were exposed at different doses of radiation (X-rays), for example, 2 Gy (common single fraction in tumour therapy) and 10 Gy (weekly fraction). We performed combined treatments with additional application of TMZ for 2 hours before irradiation and we evaluated the survival of cells and the expression and release of HMGB1 and HSP70 after single or combined treatments.

## 2. Materials and Methods

### 2.1. Cell Culture

Human glioblastoma T98G and U251MG cells were obtained from the European Collection of Cell Cultures (Porton Down, Salisbury, UK). T98G cells were cultured in Eagle's minimum essential medium (EMEM; Euroclone SpA, MI, Italy) supplemented with 10% foetal bovine serum (Sigma-Aldrich, St. Louis, MO, USA), 100 units/mL penicillin/streptomycin (Euroclone SpA, MI, Italy), 2 mM L-glutamine (Euroclone SpA, MI, Italy), and 0.01% sodium pyruvate (Sigma-Aldrich, St. Louis, MO, USA) at 37°C in an atmosphere of 5% CO_2_ and 90% humidity. U251MG cells were grown in Eagle's minimum essential medium (EMEM; Euroclone SpA, MI, Italy) supplemented with 10% foetal bovine serum (Sigma-Aldrich, St. Louis, MO, USA), 1% sodium pyruvate (Sigma-Aldrich, St. Louis, MO, USA), 2 mM glutamine (Euroclone SpA, MI, Italy), 100 U/mL of penicillin (Euroclone SpA, MI, Italy), 100 *μ*g/mL of streptomycin (Euroclone SpA, MI, Italy), and 1% nonessential amino acids (Euroclone SpA, MI, Italy) at 37°C in 5% CO_2_ and 90% humidity. Stock cultures were maintained in exponential growth as monolayers in 75 cm^2^ Corning plastic tissue-culture flasks (VWR International PBI Srl, MI, Italy).

### 2.2. Irradiation and Chemotherapic Treatments

Cells were irradiated at doses from 2 Gy to 10 Gy with X-rays at room temperature using a Raycell Mk2 (Clinica Pediatrica, IRCCS Policlinico San Matteo, Pavia, Italy) with a dose rate of 8 Gy/min. Sham irradiated cells (0 Gy) were performed as control. Before irradiation the medium was removed from the flasks and fresh medium was added in the cells.

For combined applications, cells were treated with TMZ used at concentrations of 20 *μ*M and added to the culture medium. Cells were incubated at 37°C for 2 hours with TMZ before irradiation treatment.

### 2.3. Clonogenic Survival Assay

The clonogenic assay was performed on single-cell suspension of exponentially growing cells. Cells were counted and plated in growth medium into T25 flasks and 24 h after plating they were irradiated and treated with TMZ or with combined treatments. After 10–14 days cells were stained with crystal violet solution for 6 min; colonies > 50 cells were counted. Calculation of survival fractions (SF) was performed using the equation SF = colonies counted/cells seeded × (PE/100), taking the individual plating efficiency (PE) into consideration. All experiments were repeated at least three times.

### 2.4. Immunocytochemistry Analysis

The presence and localization of danger signals (HMGB1 and HSP70) in glioblastoma cells were evaluated also with immunocytochemistry technique. Irradiated cells or combined treated cells were kept in an incubator at 37°C for 20 hours. Subsequently, cells were fixed for 10 minutes with cold 70% ethanol. As primary antibody, a polyclonal rabbit anti-HMGB1 antibody (dilution 1 : 100; Upstate, New York, USA) and a polyclonal mouse anti-HSP70 antibody (dilution 1 : 1000, BD Biosciences, NJ, USA) were used, whereas the detection was performed with the EnVision+ System-HRP (AEC) kit (Dako, Glostrup, Denmark). A semiquantitative analysis of Hsp70 and HMGB1 expression on the immunostained cells with a computer-assisted analysis was performed. In detail, the image acquisition was carried out with a digital camera (Olympus) coupled to an Olympus BX 41 optic microscope (Olympus, Milano, Italy). The digital images acquired were then processed using the ImageJ software (National Institute of Mental Health, Bethesda, Maryland, USA) that gives a measure of the intensity of the immunostaining.

### 2.5. Enzyme-Linked-Immunosorbent Assay (ELISA)

The quantification of free-proteins HSP70 and HMGB1 in supernatants was performed with an enzyme-linked-immunosorbent assay (ELISA) (resp., EIAab, China, and Uscn Life Science Inc., Houston, USA) according to the manufacturer's instructions. The minimum detectable dose of Hsp70 with our test system is less than 0.039 ng/mL and the minimum detectable dose of HMGB1 is typically less than 5.1 pg/mL

### 2.6. Statistical Analyses

Data are obtained from four independent experiments, each performed in duplicate. Statistical analyses were performed using Student's *t*-test. A *P* value of <0.05 was considered as significant (∗).

## 3. Results and Discussion

**Figure 1:**
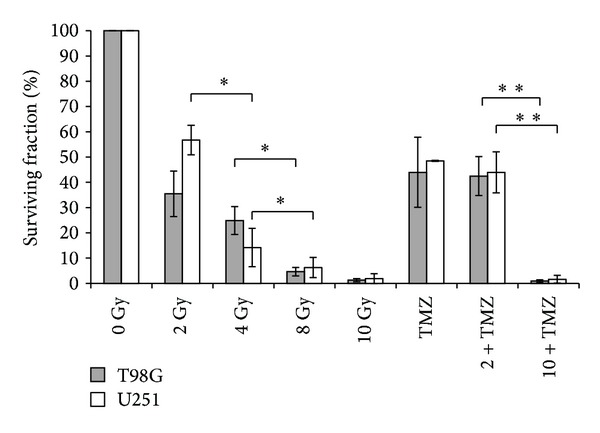
Clonogenic survival of human T98G and U251MG glioblastoma cells after single or combined treatments with radiation and temozolomide (TMZ). Means of three independent experiments ± standard deviation (∗*P* < 0.05, ∗∗*P* < 0.01).

In this work we evaluated clonogenic potential of T98G and U251MG cells after radiation treatments in comparison to radiation plus chemotherapy with TMZ. Cells treated with radiation alone showed a decrease in cell survival in a dose-dependent manner compared to control. A significant tumour growth retardation was observed, as expected, when the cells were irradiated with 8 and 10 Gy. The treatment with TMZ induced a decrease in clonogenicity compared to control but did not appear to be more effective in terms of decrease of cell survival in comparison to radiation alone. Also the combination of TMZ plus 2 or 10 Gy did not affect the percentage of surviving fraction compared to radiation alone in our experimental conditions (Figure 1).

Our data suggested that the exposure of TMZ 2 hours before irradiation did not induce an increase of radiosensitivity of T98G and U251MG cell lines. In fact, for primary malignant cell lines that express MGMT, such as T98G cells, an acute preexposure to TMZ was reported not to influence radiation cell death [10], whereas U251MG cells that are MGMT negative showed probably a radiosensitization by higher concentration of TMZ as reported by Kil and collegueas [11].

These results suggested that TMZ did not seem to have an additional effect of radiation in our experimental conditions. However, combination schedule and cell lines are important to determine an enhancement of cytotoxic effects on tumour cells and could have important implications for developing strategies to improve outcomes of patients.

Subsequently, we focus our attention on two immunogenic signals, HMGB1 and HSP70, that may lead to the activation of an immune response if released by dying cells in the microenvironment.

**Figure 2:**
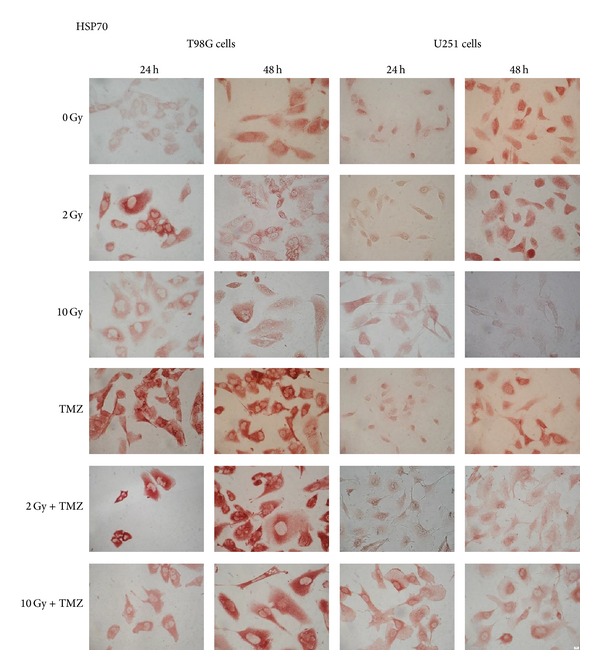
Intracellular HSP70 expression detected by immunocytochemical technique in T98G and U251MG cells after 24 and 48 hours from single or combined treatments with radiation and temozolomide (TMZ) (400x).

**Figure 3:**
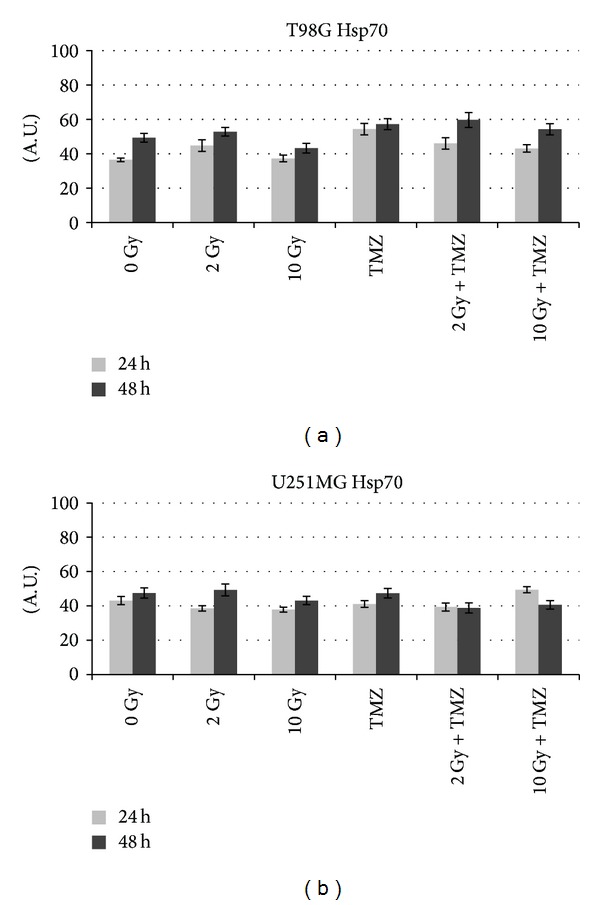
Quantitative analysis with ImageJ of Hsp70 profile in comparison with sham irradiated (0 Gy) in T98G and U251MG cells. The intensity of immunostaining is expressed as arbitrary units (A.U.) from 0 to 100.

We studied the expression and release into the medium of these free-proteins in T98G and U251MG cells after irradiation and TMZ treatments. After radiation-induced cellular stress, cells responded initially with a gradual increase of intracellular HSP70 that is produced in higher quantities with the increase of the dose up to 4 Gy and 8 Gy and is then no longer produced after 10 Gy (Tables 1 and 2, Figures 2 and 3). The increase of intracellular HSP70 continued over 48 hours and is joined to an increase in the extracellular release of the protein after high doses such as 8 and 10 Gy. These results are consistent with the externalization of HSPs that takes place in the late phases of apoptotic cell death and after a serious stress such as the one due to high doses of radiation. These results support and promote the idea about the role of HSPs as signal of damage, demonstrated to be involved in the late phases of apoptotic cell death and in stress conditions like high doses of radiation [12].

**Figure 4:**
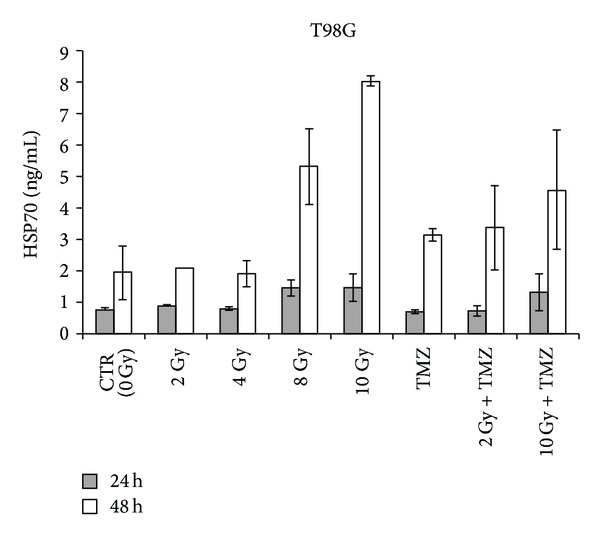
Extracellular HSP70 in T98G glioblastoma cells after 24 and 48 hours from single or combined treatments with radiation and temozolomide (TMZ) detected by ELISA. Data from 3 independent experiments are expressed as mean ± standard deviation.

T98G cells treated with TMZ alone showed an increase in the production of the protein compared to the control and irradiated cells (Table 1, Figure 2) and also an increase of the release at 48 hours compared to the control and 2 Gy treatment (Figure 4). As we can see from literature, HSP70 that is released from tumour cells undergoing chemotherapy may mediate a danger signal to the host's immune system [13, 14].

After combined treatment with TMZ and low doses of radiation, an additive effect of radiation and the drug was observed in the release of the protein in the microenvironment where HSP70 could play its immunogenic role. Extracellular HSP70 binds to high-affinity receptors on antigen presenting cells (APC), leading to activation and representation of the peptide antigen cargo by the APC. HSP70-peptide complexes coordinately activate innate immune responses and deliver antigens for representation by MHC classes I and II molecules on the APC cell surface, leading to specific antitumor immunity [15].

At higher doses (10 Gy), the effect is minor compared to radiation alone. In this case HSP70 may have a cytoprotective effect and be involved in preventing or hindering cell death. High levels of HSP70 in cytoplasm, in fact, prevent stress-induced apoptosis [16] and may induce resistance to chemotherapy and radiotherapy [15].

**Figure 5:**
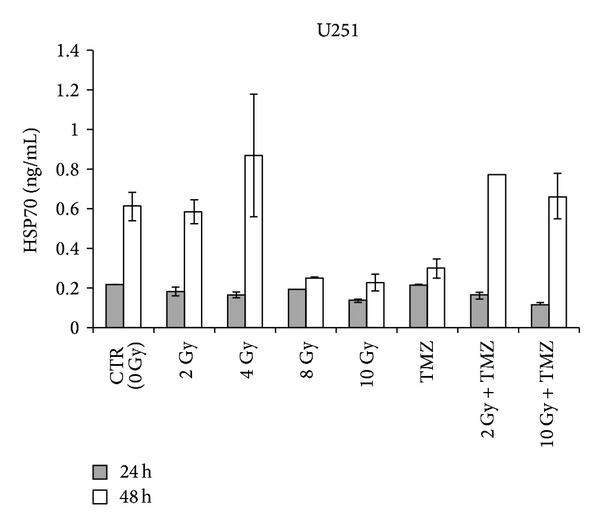
Extracellular HSP70 in U251MG glioblastoma cells after 24 and 48 hours from single or combined treatments with radiation and temozolomide (TMZ) detected by ELISA. Data from 3 independent experiments are expressed as mean ± standard deviation.

As occurred in T98G and in U87 cells analysed by us previously [17], even in U251MG the production of HSP70 increased in the first 24 hours with the increase of the dose up to 4 Gy and decreased at higher doses (Table 2, Figures 2 and 3). In this case, however, this decrease of intracellular expression is not accompanied by a release into the extracellular space (Figure 5). Probably the release occurs after a period of time longer for this cell line. In fact, after 48 hours it was detectable in the controls and after low doses. It is possible that later it occurs at higher doses and increases again at low doses.

After treatment with TMZ, there was no difference to 24 hours compared to the control or with regard to the release or the cytoplasmic expression, while at 48 hours there was a decrease of the release. The cell probably responds to TMZ with another mechanism and HSP70 pathway is not involved in TMZ-induced stress response.

The combination of TMZ and radiation induced instead an increase of the production of the protein at 24 hours followed by a release at 48 hours that was more substantial than the one after drug and radiation alone. So the drug alone did not seem to have a significant effect on the production and release of HSP70 in U251MG, probably due to the fact that this cell line has low MGMT level and TMZ tolerance [18]. However, when TMZ was combined with radiation, it induced an effect on the release of HSP70. The pathway of this protein may be triggered by combined treatments and not by single treatment with drug, probably because the radiation causes more damage pathway targeted to cell death and hence involved in the immunogenicity phenomenon, while the drug induced a mechanism more directed to the cytoprotection without activating the expression and release of damage signals.

**Figure 6:**
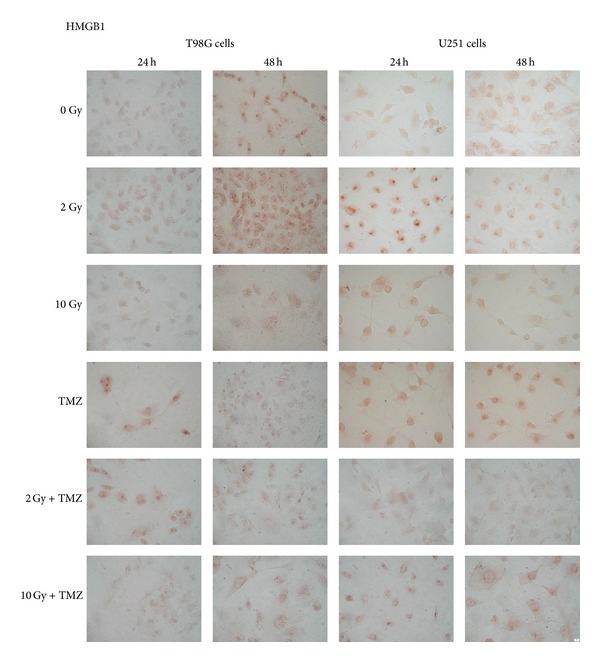
Intracellular HMGB1 expression detected by immunocytochemical technique in T98G and U251MG cells after 24 and 48 hours from single or combined treatments with radiation and temozolomide (TMZ) (400x).

**Figure 7:**
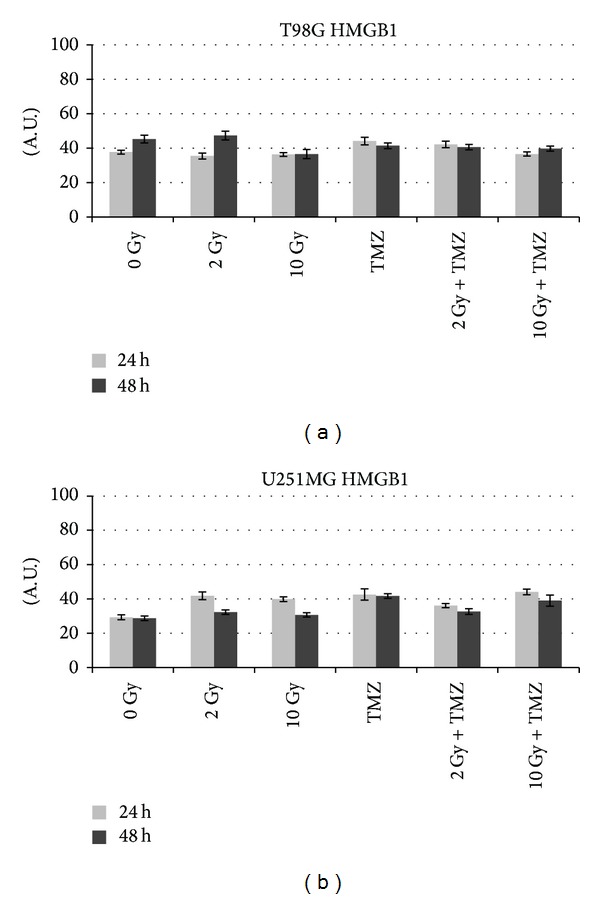
Quantitative analysis with ImageJ of HMGB1 profile in comparison with sham irradiated (0 Gy) in T98G and U251MG cells. The intensity of immunostaining is expressed as arbitrary units (A.U.) from 0 to 100.

**Figure 8:**
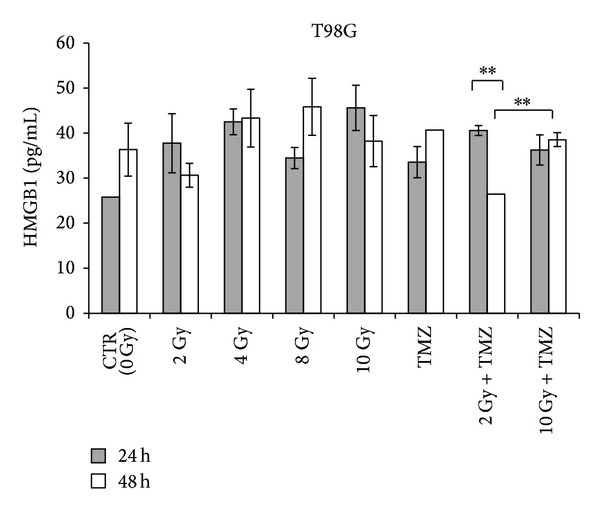
Extracellular HMGB1 in T98G glioblastoma cells after 24 and 48 hours from single or combined treatments with radiation and temozolomide (TMZ) detected by ELISA. Data from 3 independent experiments are expressed as mean ± standard deviation (∗∗*P* < 0.01).

**Figure 9:**
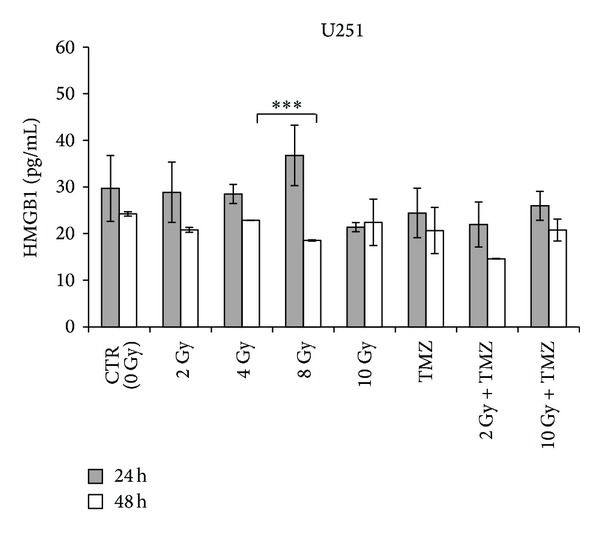
Extracellular HMGB1 in U251MG glioblastoma cells after 24 and 48 hours from single or combined treatments with radiation and temozolomide (TMZ) detected by ELISA. Data from 3 independent experiments are expressed as mean ± standard deviation (∗∗∗*P* < 0.001).

Data obtained from immunocytochemical analysis showed that in both cell lines HMGB1 had a nuclear localization in controls and in cells exposed to doses of 2 and 4 Gy (Tables 3 and 4, Figure 6). HMGB1 is ubiquitously present in the nucleus of almost all mammalian cells [19]. Within nucleus HMGB1 stabilizes nucleosomes and regulates transcription of many genes [20, 21]. At higher doses (8 Gy and 10 Gy) the expression of this protein appeared paler and the protein localization became cytoplasmatic (Figures 6 and 7). Moreover, a dose-dependent increase of HMGB1 release was observed in T98G cells after 24 hours, with the exception of the dose of 8 Gy that did not seem to induce an increase of the release (Figure 8). At this dose and this time after treatments, the cells could carry out the translocation of the protein and not its release. In U251MG the release was affected after 8 Gy exposure where it was higher compared to control. Probably this cell line needs more time to activate the release at low doses. After exposure of 10 Gy, instead, the protein was neither produced nor released (Figure 9), suggesting that this dose is that high to induce a huge damage that involves other pathways rather than the expression of DAMPs (damage-associated molecular pattern molecules). These data could demonstrate that radiation induces an increase of HMGB1 and a translocation from nucleus to cytoplasm at low doses, whereas at higher doses radiation could stimulate the release of the protein in the extracellular environment. The release of HMGB1 into the extracellular space occurs during cell death induced by radiation, both necrosis and apoptosis [22, 23], when the protein plays a role as a DAMP which activates local antigen presenting cells to become stimulatory to the immune system response [6]. The release of HMGB1 from the nucleus of dying tumour cells to their cytoplasm and subsequently to the extracellular space during cell death constitutes a crucial step in the activation of antigen presenting cells [24].

After 24 hours from treatment with 20 *μ*M of TMZ T98G, cells showed a nuclear expression of HMGB1 accompanied by an extracellular release higher than that observed in control cells (Figure 6). After 48 hours from treatment a translocation of HMGB1 in the cytoplasmic compartment was observed followed by a release of the protein that seemed to be higher than control cells but similar to the one obtained after treatment with 4 Gy (Figure 8). On the contrary, U251MG cells treated with TMZ showed no difference in the protein expression and release compared to 0 and 2 Gy treatments (Figure 9), as demonstrated previously also in U87 cells [17]. Probably the incubation time period and the drug concentration are too low to show significant changes in HMGB1 expression and localization in this cell line. Another explanation could be that TMZ did not induce a cell death type able to cause the concomitant release of this protein in this cell line. In fact, the release process may vary with cell type; generally it occurs in necrotic cells [22] but in some cell types HMGB1 could be released also by apoptotic or secondary necrotic cells [23].

Combined treatments with 2 Gy plus TMZ caused a cytoplasmatic production of HMGB1 after 24 hours, which is highly expressed in T98G and slightly in U251MG cells (Tables 3 and 4). In this condition a high release of HMGB1 compared to control was induced after short time in the first cell line, whereas in the second cell lines the values obtained are lower than those observed after single treatments. However the protein tends to be no longer produced nor released over time. On the contrary, in the condition 10 Gy plus TMZ, both after 24 and after 48 hours from treatment, a lesser presence of HMGB1 in the cytoplasm was observed due to a persistent release of the protein. At 10 Gy plus TMZ the situation is quite similar to that obtained in the single although the release of HMGB1, after 24 hours, is different for each cell line: lower in T98G and slightly greater in U251MG in comparison to that obtained by 10 Gy alone (Figures 8 and 9). Results obtained from combined treatments did not suggest a beneficial use of combined treatments in comparison with radiation alone to activate signalling of immunogenic proteins able to stimulate an antitumoral response.

## 4. Conclusions

The success of some chemo- and radiotherapeutic regimens could rely on the induction of immunogenic tumour cell death and on the induction of anticancer immune response. In glioblastoma conventional cancer treatments are destined to fail because of dormant micrometastases or tumour (stem) cells resistant to therapy and able to induce relapse and therapeutic failure. One possible strategy consists in stimulating the immunogenicity of tumour cells, resulting in the expose, release, or active specific immunogenic factors and molecules that change microenvironment and deliver a stimulatory signal to the immune system [12]. Only few data exist dealing with the immune sensitizing effects of TMZ when added to radiation in human glioma and the identity of danger signals are still not better known in this kind of tumours.

In this work we studied two danger signals HSP70 and HMGB1 that are expressed and released in response to single or combined treatments of radiation and temozolomide in glioblastoma cells.

In particular, our data suggested that HSP70 translocates from cytoplasm to extracellular environment after an increase of radiation dose. When cell undergoes cellular stress, such as exposure to radiation, many HSPs are overexpressed in the cytoplasm. Some of these proteins such as HSP70 are also able to move from cytoplasm to plasmatic membrane and subsequently outside the cells in order to carry out a potent immune-stimulatory activity [25]. Our results reported a HMGB1 translocation from the nucleus to the cytoplasm and subsequent release into the extracellular space after irradiation in glioblastoma cells. It is likely that HMGB1 released into the microenvironment as a result of tumour cells induced by radiation facilitates the activation of dendritic cells within the tumour [9]. However, the increase of HMGB1 in the extracellular space could drive cancer progression because of its activity as autocrine factor capable of promoting the growth and migration of tumour cells [26]. Further studies should be carried on to better understand the mechanism of action of HMGB1 and HSP70 on glioblastoma cells and on immune system cells to activate an immune response against tumour.

We also could state that TMZ had limited effectiveness in activating HMGB1 and HSP70 signalling and an adjuvant effect in their expression and release, instead, was observed in some combined treatments, but it depends on schedule, cell line, and timing, as reported by Chalmers and colleagues [27]. A big challenge in tumour therapy is, therefore, to identify the most beneficial combination and chronology of multiple treatment options to contribute to the improvement of the therapeutic outcome. Moreover, future work should focus on the modification of brain microenvironment through the enhancing or triggering of DAMPs signalling that is necessary to stimulate antitumour response and elicit tumour regression and long-term immunological memory.

## Figures and Tables

**Table 1:** Intracellular expression of HSP70 in T98G cells after 24 and 48 hours from single or combined treatments with radiation and temozolomide (TMZ).

HSP70	0 Gy	2 Gy	4 Gy	8 Gy	10 Gy	TMZ	2 Gy + TMZ	10 Gy + TMZ
24 h	0/+	++/+++	++	+/++	+	+++	++/+++	+/++
48 h	++	+/++	++/+++	++/+++	+	+++	+++	++/+++

0: no expression of protein; +: low expression of protein; ++: moderate expression of protein; +++: high expression of protein.

**Table 2:** Intracellular expression of HSP70 in U251MG cells after 24 and 48 hours from single or combined treatments with radiation and temozolomide (TMZ).

HSP70	0 Gy	2 Gy	4 Gy	8 Gy	10 Gy	TMZ	2 Gy + TMZ	10 Gy + TMZ
24 h	+/++	+/++	++/+++	++	++	+/++	++	+++
48 h	+++	++/+++	++	++/+++	0/+	+++	+/++	++

0: no expression of protein; +: low expression of protein; ++: moderate expression of protein; +++: high expression of protein.

**Table 3:** Intracellular expression of HMGB1 in T98G cells after 24 and 48 hours from single or combined treatments with radiation and temozolomide (TMZ).

HMGB1	0 Gy	2 Gy	4 Gy	8 Gy	10 Gy	TMZ	2 Gy + TMZ	10 Gy + TMZ
24 h	+ (n)	+/++ (n)	++ (n)	+/++ (c)	+/++ (c)	+++ (n)	++/+++ (c)	+ (c)
48 h	++/+++ (n/p)	+++ (n)	++/+++ (n)	+/++ (c)	+ (c)	+/++ (p)	+/++ (c)	+/++ (c)

0: no expression of protein; +: low expression of protein; ++: moderate expression of protein; +++: high expression of protein; n: nuclear expression; p: perinuclear expression; c: cytoplasmatic expression.

**Table 4:** Intracellular expression of HMGB1 in U251MG cells after 24 and 48 hours from single or combined treatments with radiation and temozolomide (TMZ).

HMGB1	0 Gy	2 Gy	4 Gy	8 Gy	10 Gy	TMZ	2 Gy + TMZ	10 Gy + TMZ
24 h	+/++ (n)	++/+++ (p)	+ (n)	+++ (c)	+/++ (c)	++ (n)	+ (c)	+/++ (c)
48 h	+/++ (n)	++ (p)	+++ (p)	++/+++ (c)	+ (c)	++/+++ (p)	+ (c)	++ (p)

0: no expression of protein; +: low expression of protein; ++: moderate expression of protein; +++: high expression of protein; n: nuclear expression; p: perinuclear expression; c: cytoplasmatic expression.

## References

[1] Virrey JJ, Golden EB, Sivakumar W (2009). Glioma-associated endothelial cells are chemoresistant to temozolomide. *Journal of Neuro-Oncology*.

[2] Ichimura K, Ohgaki H, Kleihues P, Collins VP (2004). Molecular pathogenesis of astrocytic tumours. *The Journal of Neuro-Oncology*.

[3] Han W, Chen S, Yu KN, Wu L (2010). Nitric oxide mediated DNA double strand breaks induced in proliferating bystander cells after *α*-particle irradiation. *Mutation Research/Fundamental and Molecular Mechanisms of Mutagenesis*.

[4] Zitvogel L, Apetoh L, Ghiringhelli F, André F, Tesniere A, Kroemer G (2008). The anticancer immune response: indispensable for therapeutic success?. *The Journal of Clinical Investigation*.

[5] Fonseca C, Dranoff G (2008). Capitalizing on the immunogenicity of dying tumor cells. *Clinical Cancer Research*.

[6] Kono H, Rock KL (2008). How dying cells alert the immune system to danger. *Nature Reviews Immunology*.

[7] Hodge JW, Guha C, Neefjes J, Gulley JL (2008). Synergizing radiation therapy and immunotherapy for curing incurable cancers: opportunities and challenges. *Oncology*.

[8] Hagemann T, Balkwill F, Lawrence T (2007). Inflammation and cancer: a double-edged sword. *Cancer Cell*.

[9] Curtin JF, Liu N, Candolfi M (2009). HMGB1 mediates endogenous TLR2 activation and brain tumor regression. *PLoS Medicine*.

[10] Wedge SR, Porteous JK, Glaser MG, Marcus K, Newlands ES (1997). In vitro evaluation of temozolomide combined with X-irradiation. *Anti-Cancer Drugs*.

[11] Kil WJ, Cerna D, Burgan WE (2008). In vitro and in vivo radiosensitization induced by the DNA methylating agent temozolomide. *Clinical Cancer Research*.

[12] Kepp O, Galluzzi L, Martins I (2011). Molecular determinants of immunogenic cell death elicited by anticancer chemotherapy. *Cancer and Metastasis Reviews*.

[13] Calderwood SK, Theriault JR, Gong J (2005). Message in a bottle: role of the 70-kDa heat shock protein family in anti-tumor immunity. *European Journal of Immunology*.

[14] Ciocca DR, Rozados VR, Cuello Carrion FD, Gervasoni SI, Matar P, Scharovsky OG (2003). Hsp25 and Hsp70 in rodent tumors treated with doxorubicin and lovastatin. *Cell Stress and Chaperones*.

[15] Gehrmann M, Radons J, Molls M, Multhoff G (2008). The therapeutic implications of clinically applied modifiers of heat shock protein 70 (Hsp70) expression by tumor cells. *Cell Stress and Chaperones*.

[16] Brondani Da Rocha A, Regner A, Grivicich I (2004). Radioresistance is associated to increased Hsp70 content in human glioblastoma cell lines. *International Journal of Oncology*.

[17] Paolini A, Pasi F, Facoetti A (2011). Cell death forms and HSP70 expression in U87 cells after ionizing radiation and/or chemotherapy. *Anticancer Research*.

[18] van Rijn J, Heimans JJ, van den Berg J, van der Valk P, Slotman BJ (2000). Survival of human glioma cells treated with various combination of temozolomide and X-rays. *International Journal of Radiation Oncology, Biology, Physics*.

[19] Ellerman JE, Brown CK, de Vera M (2007). Masquerader: high mobility group box-1 and cancer. *Clinical Cancer Research*.

[20] Park JS, Arcaroli J, Yum HK (2003). Activation of gene expression in human neutrophils by high mobility group box 1 protein. *The American Journal of Physiology: Cell Physiology*.

[21] Stros M, Ozaki T, Bacikova A, Kageyama H, Nakagawara A (2002). HMGB1 and HMGB2 cell-specifically down-regulate the p53- and p73-dependent sequence-specific transactivation from the human Bax gene promoter. *The Journal of Biological Chemistry*.

[22] Rovere-Querini P, Capobianco A, Scaffidi P (2004). HMGB1 is an endogenous immune adjuvant released by necrotic cells. *EMBO Reports*.

[23] Bell CW, Jiang W, Reich CF, Pisetsky DS (2006). The extracellular release of HMGB1 during apoptotic cell death. *American Journal of Physiology—Cell Physiology*.

[24] Apetoh L, Ghiringhelli F, Tesniere A (2007). The interaction between HMGB1 and TLR4 dictates the outcome of anticancer chemotherapy and radiotherapy. *Immunological Reviews*.

[25] Tesniere A, Apetoh L, Ghiringhelli F (2008). Immunogenic cancer cell death: a key-lock paradigm. *Current Opinion in Immunology*.

[26] Bassi R, Giussani P, Anelli V (2008). HMGB1 as an autocrine stimulus in human T98G glioblastoma cells: role in cell growth and migration. *Journal of Neuro-Oncology*.

[27] Chalmers AJ, Ruff EM, Martindale C, Lovegrove N, Short SC (2009). Cytotoxic effects of TMZ and radiation are additive- and schedule-dependent. *International Journal of Radiation Oncology Biology Physics*.

